# Identification and validation of colorectal neoplasia-specific methylation biomarkers based on CTCF-binding sites

**DOI:** 10.18632/oncotarget.23172

**Published:** 2017-12-11

**Authors:** Jiang Liu, Zhaoli Ding, Guimei Li, Li Tang, Yu Xu, Huayou Luo, Jinhua Yi, Youwang Lu, Rui Mao, Qiong Nan, Li Ren, Tong Zhang, Kunhua Wang

**Affiliations:** ^1^ Department of Reproduction and Genetics, The First Affiliated Hospital of Kunming Medical University, Kunming 650032, Yunnan, China; ^2^ Public Technical Service Center, Kunming Institute of Zoology, Chinese Academy of Sciences, Kunming 650032, Yunnan, China; ^3^ Kunming Biological Diversity Regional Center of Large Apparatus and Equipments, Chinese Academy of Sciences, Kunming 650032, Yunnan, China; ^4^ Department of Gastrointestinal Surgery, The First Affiliated Hospital of Kunming Medical University, Yunnan Institute of Digestive Disease, Kunming 650032, Yunnan, China; ^5^ School of Stomatology, Kunming Medical University, Kunming 650500, Yunnan, China; ^6^ Department of Gastroenterology, The First Affiliated Hospital of Kunming Medical University, Yunnan Institute of Digestive Disease, Kunming 650032, Yunnan, China; ^7^ The First People’s Hospital of Yunnan Province, Kunming 650031, Yunnan, China

**Keywords:** colorectal cancer, early-detection, CTCF-binding site, DNA methylation, biomarker

## Abstract

To date, the sensitivity of currently available biomarkers based on the methylation of gene promoters is suboptimal for detecting adenomas and early-stage colorectal cancer (CRC). We aimed to develop biomarkers with methylated DNA binding sites of the multifunctional transcriptional factor CTCF for early detection of CRC. Using combined analyses of genome-wide occupation and the methylation profile of CTCF-binding sites, we identified candidate CTCF-binding sites. Then, we applied methylation-sensitive high-resolution melting (MS-HRM) and mass spectrometry analysis to screen and validate these candidate sites in diverse sample sets. We identified a set of colorectal neoplasia-specific biomarkers with robust performance. The top five biomarkers were selected and recommended for early detection of colorectal neoplasia. All of the five novel biomarkers exhibited a more robust discriminatory performance than that by *BMP3* and *NDRG4*, two currently acknowledged robust methylation biomarkers. When the five new biomarkers were considered as a marker panel and tumor-positive was defined as having two or more (of the five) positive biomarkers, the marker panel could achieve a sensitivity of 91.67% for adenomas, 97.44% for Stage I CRC, 94.06% for Stage II CRC, 93.62% for Stage III CRC, and 93.54% for total colorectal tumors with a specificity of 94.05%. To our knowledge, this is the first study for colorectal neoplasia-specific methylation biomarkers based on CTCF-binding sites. Using a similar strategy, CTCF-binding sites could be potentially developed into biomarkers for other tumors. In summary, this study opens a new area in developing biomarkers for tumor prevention and treatment.

## INTRODUCTION

Colorectal cancer (CRC) is the third most commonly diagnosed cancer in males and second in females [1]. DNA methylation, which is one of the most important mechanisms involved in gene and microRNA expression regulation and in alternative gene splicing, plays important roles in the early stage of cancer [2]. Because it is stable and easily detected qualitatively or quantitatively, DNA methylation has been considered as the most promising diagnostic marker for early detection of cancer [3]. Over the recent years, many DNA methylation markers have been proposed as useful early detection markers for CRC [2, 4–14]. For example, the development of blood-based cancer detection tests improves patient compliance, thereby increasing the incidence of cancer detection at earlier stages. Currently, the most established methylated DNA blood biomarker is methylated septin 9 (*SEPT9*) [15, 16]. *SEPT9* is now commercially offered as a blood-based screening test in various assays. However, methylated *SEPT9* has a limited sensitivity for the detection of advanced adenomas (11%), underscoring the need for further improvement of this test for its implementation in population-based screening of colorectal neoplasia [17]. In addition, a stool DNA test (sDNA test), which includes quantitative molecular assays for *KRAS* (kirsten rat sarcoma viral oncogene) mutations, aberrant *NDRG4* (N-myc downstream-regulated 4) and *BMP3* (bone morphogenetic protein 3) methylation, and a hemoglobin immunoassay [5, 6, 13], has culminated in the development of an FDA approved, clinically available stool-based CRC screening test [2]. However, its sensitivity for detecting advanced precancerous lesions was only 42.4% (*P* < 0.001) [13]. In summary, although the impressive accuracy for CRC detection has been achieved, both serum-based DNA test and stool-based DNA test are currently less sensitive for CRC precursor lesions, particularly for adenomas [4-6, 13]. Therefore, urgent development of novel and robust detective biomarkers for the early detection of adenomas and CRC is required. Previously reported methylation biomarkers were primarily based on the methylation of gene promoters, but this study aimed to develop biomarkers with DNA binding sites of a multifunctional transcriptional factor CTCF (CCCTC-binding factor) for early detection of CRC. CTCF is a versatile transcription regulator that is ubiquitously expressed and evolutionarily conserved from the fruit fly to humans [18]. CTCF contains a highly conserved DNA-binding domain with 11 zinc fingers, and it binds to different DNA sequences by the combinatorial use of 11-zinc fingers to play a key role in many chromatin insulation events and epigenetic processes [19–26]. Despite its obvious importance, to our knowledge, there is no report considering CTCF-binding sites as biomarkers for the early detection of tumors. We considered CTCF-binding sites as potential biomarkers for early detection of CRC because of the following reasons: (i) CTCF-binding sites are widespread in human genome and CTCF occupancy at its binding sites is generally methylation-sensitive [27–29]. (ii) Aberrant DNA methylation of CTCF-binding sites has also been connected with multiple malignancies [21, 30–35]. (iii) CTCF is involved in the maintenance of genomic methylation patterns [36, 37]. (iv) Immortalized cancer cell lines contain cell type-specific CTCF-binding pattern, which is significantly associated with differential methylation status [29]. Therefore, the methylation status of CTCF-binding sites is a potentially ideal biomarker for early detection of CRC for tumor prevention and treatment.

By genome-wide identification of candidate CTCF-binding sites using the combined analyses of genome-wide occupation and methylation profile of CTCF-binding site with published genome-wide data, followed by stepwise screening and verification using methylation-sensitive high-resolution melting (MS-HRM) and mass spectrometry analysis, this study aimed to (1) determine the performance of CTCF-binding sites in discriminating tumor tissues from normal tissues, (2) assess the potential of CTCF-binding sites as biomarkers for early-detection of CRC, and (3) provide a valuable reference to develop new biomarkers for the prevention and treatment of other tumors.

## RESULTS

### MS-HRM analysis identified 23 CTCF-binding sites with high tumor specificity from the 121 candidate CTCF-binding sites

**Figure 1:**
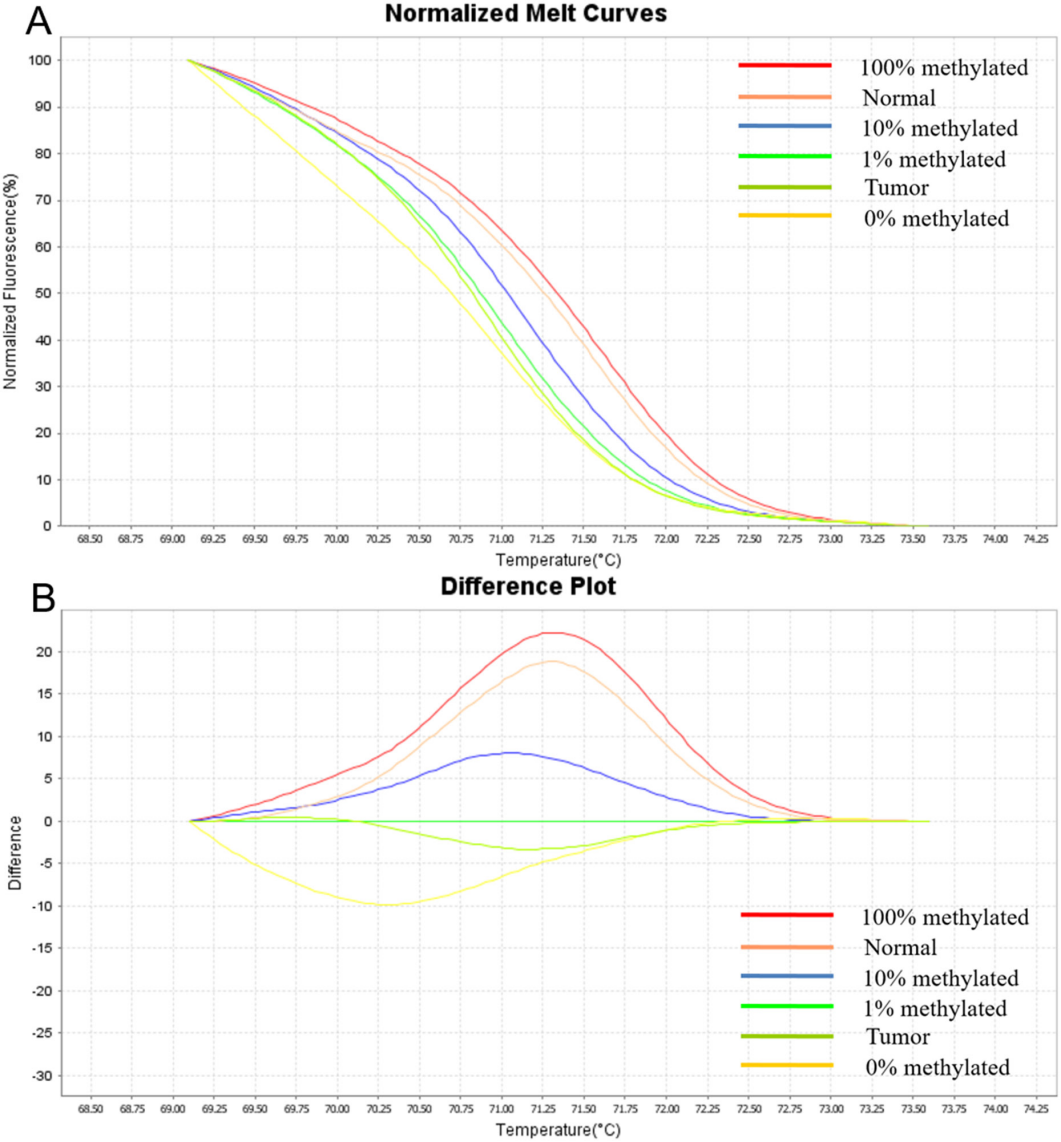
**(A)** Normalized melting profile. **(B)** Difference plots. The HRM standard melting curve was derived from five samples, with the following ratios of methylated DNA: 0, 1, 10, and 100% methylation. At the annealing temperature of 58°C, methylation levels as low as 1% could be easily detected. As shown in the figure, the methylation level of a representative CTCF-binding site in a tumor tissue is between 0–1%, and its methylation level in the matched normal counterpart is between 10–100%, indicating that the methylation level of this CTCF-binding site is significantly lower in tumor tissues than that in matched normal counterparts.

By using different dilutions of fully methylated DNA against unmethylated DNA, with ratios of methylated DNA as 0, 1, 10, and 100% methylation, we showed that methylation levels as low as 1% could be easily detected at the annealing temperature of 58°C by MS-HRM analysis (Figure 1). For the 121 candidate CTCF-binding sites, MS-HRM analysis identified 23 CTCF-binding sites with methylation profiles highly specific in tumor tissues and were, thus, considered as potential candidates for colorectal neoplasia biomarkers (Supplementary Table 1); the remaining 98 CTCF-binding sites were eliminated because they did not exhibit impressive tumor-specific methylation profiles. The methylation pattern of these 23 CTCF-binding sites was specific in 64%–94% of the tested tumor tissues. Among the 23 CTCF-binding sites, methylation levels of 16 sites were significantly higher in tumor tissues than those in matched normal counterparts (T > N). For the remaining seven sites, methylation levels were significantly lower in tumor tissues than those in matched normal counterparts (T < N).

### Mass spectrometry analysis further screened out 5 final biomarkers with impressive detection accuracy from the 23 CTCF-binding sites

To accurately quantify the DNA methylation differences between normal and tumor tissues, to evaluate the performance of different CTCF-binding sites in discriminating tumor tissues from normal tissues, the methylation state of the 23 tumor-specific CTCF-binding sites identified by MS-HRM analysis was further determined by mass spectrometry analysis in 20 pairs of normal and tumor tissues. Twenty tumor tissues comprised seven adenomas, six Stage I CRC, and seven Stage II CRC.

**Figure 2:**
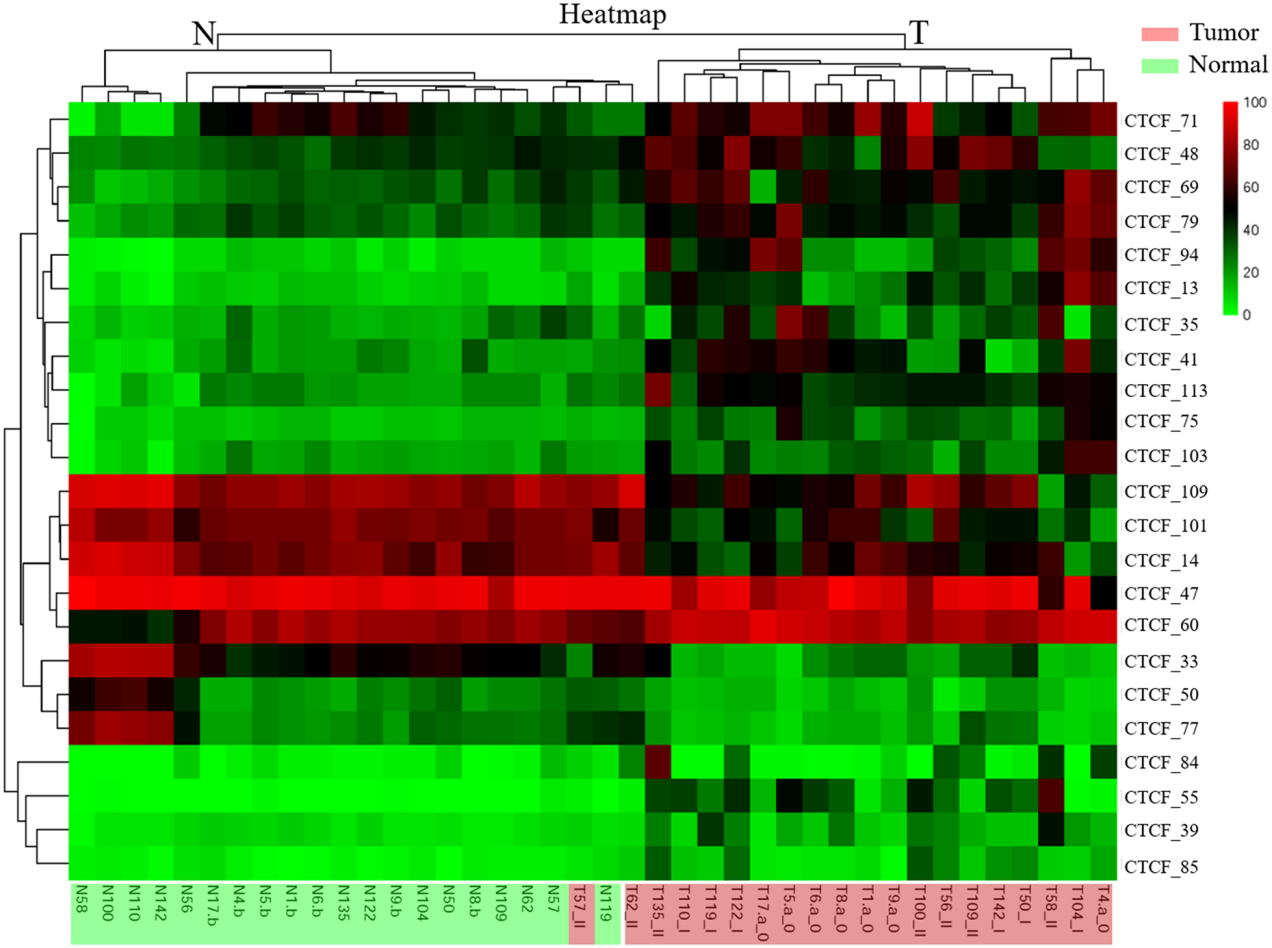
Hierarchical clustering of all 40 colorectal tissue samples and 23 tumor-specific CTCF-binding sites identified by MS-HRM analysis Columns, patient samples; rows, tumor-specific CTCF-binding sites. Column annotations include the names of the CTCF-binding sites, row annotations include the names of the sample (N represents normal tissue; T represents tumor tissue) and tumor stages for tumor tissues (0 represents adenomas; I represents Stage I; II represents Stage II). The class information was unknown to the clustering algorithm. The average degree of methylation of each CTCF-binding site in each sample is represented by the decadic logarithm of the methylation proportion ranging from 0% methylated alleles (green) to methylation of all alleles (red). There are two main tissue clusters labeled as N and T. Cluster N consists of 18 normal tissues and two tumor tissues. Cluster T is composed of the remaining 18 tumor tissues.

To determine the clustering profile in the overall methylation patterns of the samples and CTCF-binding sites, a hierarchical two-dimensional unsupervised clustering was performed between a set of 23 tumor-specific CTCF-binding sites and a set of 40 colorectal tissue samples (Figure 2). All of the 40 colorectal samples were divided into two clusters (cluster N and cluster T). Cluster N consisted of 20 normal tissues and two tumor tissues. Cluster T included the remaining 18 tumor tissues. In addition, mass spectrometry analysis confirmed methylation patterns of the 23 CTCF-binding sites identified by MS-HRM analysis, indicating that methylation levels of 16 sites were significantly higher in tumor tissues than those in the matched normal counterparts and the methylation levels of the remaining seven sites were significantly lower in tumor tissues than those in matched normal counterparts. The clustering results clearly showed that the 23 CTCF-binding sites could discriminate tumor tissues from normal colorectal tissues. We will next try to determine the ability of each CTCF-binding site to discriminate tumor tissues from normal tissues.

To find the most robust candidate biomarkers for the early detection of colorectal neoplasia, we used ROC curves to quantify the accuracy and robustness of each CTCF-binding site. In this analysis, the negative class consisted of 20 normal colorectal samples, and the positive class included 20 colorectal tumor samples. The discriminatory powers of 23 CTCF-binding sites are summarized in Table 1. Despite the relatively lower sample number in this analysis, the discriminatory powers of the 18 CTCF-binding sites were statistically significant (*P* < 0.0001). Nineteen sites exhibited a very robust discriminatory power with an AUC of >0.8. The sensitivity of these strong markers ranged between 50% and 97.37% at a specificity level of 95%. According to the discriminatory power of individual CTCF-binding sites and the manual review of the discriminatory power for samples from early stage colorectal neoplasia (adenomas and Stage I CRC), ten CTCF-binding sites were selected for further screening (marked in bold in Table 1). Of these ten CTCF-binding sites, the methylation levels of five sites in tumor tissues were significantly lower than those in normal tissues.

**Table 1:** Single-marker performance of the 23 candidate CTCF-binding sites

CTCF site	AUC	*P*	Sensitivity at 95% specificity
**CTCF_13**	**0.997**	<0.0001	97.37 [82.46,100.0]
**CTCF_113**	**0.995**	<0.0001	95.00 [75.00,100.0]
CTCF_75	0.993	<0.0001	95.95 [75.99,100.0]
**CTCF_55**	**0.984**	<0.0001	80.00 [50.00,95.00]
**CTCF_79**	**0.961**	<0.0001	80.00 [55.00,95.00]
**CTCF_94**	**0.956**	<0.0001	85.00 [60.00,95.00]
**CTCF_33**	**0.954**	<0.0001	84.21 [54.49,94.74]
CTCF_69	0.946	<0.0001	89.47 [62.38,100.0]
**CTCF_14**	**0.92**	<0.0001	68.42 [36.84,84.21]
**CTCF_101**	**0.917**	<0.0001	71.11 [43.97,88.89]
CTCF_85	0.916	<0.0001	67.50 [40.00,87.50]
CTCF_39	0.907	<0.0001	75.00 [55.00,90.00]
CTCF_103	0.891	<0.0001	50.00 [25.00,80.00]
CTCF_109	0.879	<0.0001	70.00 [45.00,85.10]
**CTCF_50**	**0.866**	<0.0001	63.16 [36.84,84.21]
CTCF_41	0.838	<0.0001	68.42 [47.37,89.47]
**CTCF_77**	**0.828**	<0.0001	65.00 [45.00,85.00]
CTCF_60	0.826	<0.0001	55.00 [30.00,80.00]
CTCF_48	0.809	0.0001	63.16 [36.84,82.88]
CTCF_35	0.781	0.0012	38.33 [03.52,65.88]
CTCF_71	0.754	0.0012	36.84 [10.53,57.89]
CTCF_47	0.724	0.0065	25.00 [0.77,45.00]
CTCF_84	0.63	0.1561	35.00 [15.00,60.00]

**NOTE:** Shown is the AUC, P value, and sensitivity at 95% specificity (median value plus 90% confidence interval estimated by bootstrapping) for each marker.

To reinforce the discriminatory power of our methylation biomarkers for adenomas, the methylation level of the 10 CTCF-binding sites was further determined in 90 samples by increasing the proportion of adenoma samples (20 normal tissues, 57 adenomas, six Stage I CRC, and seven Stage II CRC) by mass spectrometry analysis. The discriminatory powers of these 10 sites are summarized in Table 2. In this analysis, all of the 10 sites were statistically significant (*P* < 0.0001) and exhibited a very strong class separation with an AUC of ≥0.85. The sensitivity of these strong markers ranged between 44.64% and 88.89% at a specificity level of 95%. We strove to identify a panel as small as possible that would accurately identify colorectal tumors in the early stage to provide the most cost-effective screening method for early detection of colorectal tumors. Therefore, according to their discriminatory performance (AUC value), five CTCF-binding sites (marked in bold in Table 2) were selected as final biomarkers for further validation in a large sample set. Among these five CTCF-binding sites, the methylation level of one site (CTCF_33) in the tumor tissues was significantly lower than that in normal tissues. For the remaining four sites, methylation levels in tumor tissues were significantly higher than those in normal tissues. The discriminatory powers of these five sites were very strong with an AUC of ≥0.916, and the sensitivity ranged between 73.08% and 88.89% at a specificity level of 95%.

**Table 2:** Single-marker performance of the 10 candidate CTCF-binding sites

CTCF site	AUC	*P*	Sensitivity at 95% specificity
**CTCF_113**	**0.977**	<0.0001	88.89 [68.45,96.83]
**CTCF_13**	**0.953**	<0.0001	80.08 [54.75,96.95]
**CTCF_33**	**0.943**	<0.0001	80.47 [66.85,89.24]
**CTCF_55**	**0.934**	<0.0001	80.77 [65.58,90.68]
**CTCF_94**	**0.916**	<0.0001	73.08 [60.38,82.50]
CTCF_79	0.906	<0.0001	74.46 [61.17,83.69]
CTCF_101	0.881	<0.0001	44.64 [28.57,75.00]
CTCF_14	0.868	<0.0001	59.38 [37.81,75.62]
CTCF_77	0.863	<0.0001	74.23 [59.50,84.69]
CTCF_50	0.85	<0.0001	54.71 [38.26,74.98]

### Discriminatory performance of the five final biomarkers was reconfirmed with a large sample set by mass spectrometry analysis

#### Individual marker performance

The discriminatory performance of the five CTCF-binding sites selected as final biomarkers were further validated in a large sample set by mass spectrometry. The methylation level of these five CTCF-binding sites was examined in a large sample set (379 samples in total) with a negative class of 84 colorectal-derived normal tissues. The positive class was composed of 295 colorectal-derived tumor tissues from adenomas (108), Stage I CRC (39), Stage II CRC (101), and Stage III CRC (47). We analyzed the individual marker performance of the five final sites in adenomas, Stage I CRC, Stage II CRC, Stage III CRC, and total colorectal-derived tumor tissues, respectively (Table 3). As shown in Table 3, regardless of the adenoma, Stage I CRC, Stage II CRC, or Stage III CRC, all of the five markers were statistically significant (*P* < 0.0001) and showed a very strong individual performance with an AUC of ≥0.853. The sensitivity of these five markers ranged between 57.95% and 96.84% at a specificity level of 95%. In particular, for adenomas, all of the five markers showed a very strong individual performance with an AUC of ≥0.917, and the sensitivity ranged between 73.52% and 82.62% at a specificity level of 95%. All markers exhibited a very strong individual performance with an AUC of ≥0.891 for all colorectal-derived tumor tissues, and the sensitivity ranged between 68.68% and 87.14% at a specificity level of 95%.

**Table 3:** The discriminatory performance of the five final CTCF-binding sites in the large sample set

CTCF site	Adenoma (N=108)			Stage I (N=39)			Stage II (N=101)			Stage III (N=47)			Total (N=295)		
	AUC	*P*	Sensitivity at 95% specificity	AUC	*P*	Sensitivity at 95% specificity	AUC	*P*	Sensitivity at 95% specificity	AUC	*P*	Sensitivity at 95% specificity	AUC	*P*	Sensitivity at 95% specificity
CTCF_33	0.93	<0.0001	80.51 [68.94,88.37]	0.9	<0.0001	67.47 [49.41,83.45]	0.9	<0.0001	57.59 [46.78,69.11]	0.9	<0.0001	65.22 [47.83,80.43]	0.9	<0.0001	68.68 [60.17,76.25]
CTCF_55	0.93	<0.0001	73.52 [64.75,82.33]	1	<0.0001	95.13 [82.07,100.0]	1	<0.0001	88.87 [59.14,95.88]	0.9	<0.0001	80.77 [63.92,91.52]	0.9	<0.0001	84.66 [76.19,88.87]
CTCF_94	0.92	<0.0001	79.44 [70.09,86.61]	1	<0.0001	83.59 [61.83,92.31]	0.9	<0.0001	74.46 [63.37,82.24]	0.9	<0.0001	74.04 [47.92,87.41]	0.9	<0.0001	77.41 [70.09,84.04]
CTCF_113	0.92	<0.0001	82.62 [72.41,89.91]	1	<0.0001	92.92 [81.76,98.57]	1	<0.0001	89.19 [81.98,94.63]	0.9	<0.0001	89.36 [76.60,97.87]	0.9	<0.0001	85.17 [79.23,90.01]
CTCF_13	0.93	<0.0001	79.79 [70.56,87.08]	1	<0.0001	96.84 [77.68,100.0]	1	<0.0001	88.06 [80.43,93.10]	1	<0.0001	87.66 [73.66,95.94]	1	<0.0001	87.14 [81.77,91.31]

We next compared the discriminatory power of these five new markers with two currently acknowledged robust methylation biomarkers for the early detection of colorectal tumors (*BMP3* and *NDRG4*) in the same sample set. *BMP3* and *NDRG4* are methylation biomarkers of the sDNA test, which has culminated in the development of an FDA-approved and clinically available stool-based CRC screening test [2, 13]. One hundred and twenty colorectal samples (including 58 normal tissues, 46 adenomas, four Stage I CRC, eight Stage II CRC, and four Stage III CRC) were selected from the large sample set, and the methylation status of *BMP3* and *NDRG4* was determined by mass spectrometry. ROC curves were used to compare the accuracy and robustness of these seven markers (five new markers and *BMP3* and *NDRG4*; Table 4). Although all the seven markers were statistically significant (*P* < 0.0001), each of the five new markers exhibited a more robust discriminatory performance than that by *BMP3* or *NDRG4*. As shown in Table 4, the five new markers had an AUC of ≥0.89, and the sensitivity ranged between 73.83% and 86.72% at a specificity level of 90%, whereas *BMP3* had an AUC of 0.79 and the sensitivity was 48.56% at a specificity level of 90% and *NDRG4* had an AUC of 0.88 with the sensitivity of 69.56% at a specificity level of 90%. In particular, the sensitivity of four of the five new markers was over 81% at a specificity level of 90%, but both *BMP3* and *NDRG4* exhibited less than 70% sensitivity, which is a further indication that the discriminatory accuracy of our new markers is significantly higher than that of *BMP3* or *NDRG4*.

**Table 4:** Comparison of new markers with existing markers

Marker	AUC	*P*	Sensitivity at 90% specificity
CTCF_33	0.9	<0.0001	81.69 [61.51,93.23]
CTCF_55	0.92	<0.0001	81.02 [68.86,88.35]
CTCF_94	0.89	<0.0001	73.83 [56.97,85.06]
CTCF_113	0.92	<0.0001	86.72 [73.32,95.00]
CTCF_13	0.93	<0.0001	81.64 [67.75,92.07]
BMP3	0.79	<0.0001	48.56 [32.06,61.96]
NDRG4	0.88	<0.0001	69.56 [50.94,82.21]

**NOTE:** Shown is the AUC, P value, and sensitivity at 90% specificity (median value plus 90% confidence interval estimated by bootstrapping) for each marker.

#### Marker panel performance

When we considered the five final CTCF-binding sites as a panel of markers for the early-detection of colorectal tumors, the discriminatory performance improved significantly compared to that of the best single marker. Marker panel performance for different colorectal tumor stages is summarized in Table 5. When tumor-positive was defined as having two or more (of the five) positive CTCF-binding sites, the marker panel yielded the best sensitivity and specificity (Table 5). In this situation, the sensitivity of the marker panel was 91.67% for adenomas, 97.44% for Stage I CRC, 94.06% for Stage II CRC, 93.62% for Stage III CRC, and 93.54% for total colorectal tumors at a specificity level of 94.05%.

**Table 5:** Marker panel performance

No. of CTCF site	Sensitivity (%)					Specificity (%)
	Adenoma	Stage I	Stage II	Stage III	Total	
≥1	96.30	97.44	98.02	95.74	97.28	79.76
**≥2**	**91.67**	**97.44**	**94.06**	**93.62**	**93.54**	**94.05**
≥3	85.19	92.31	91.09	87.23	89.8	97.62
≥4	74.07	84.62	75.25	65.96	74.49	100
5	50.93	56.41	40.59	51.06	47.96	100

## DISCUSSION

By genome-wide identification of candidate CTCF-binding sites using the combination of genome-wide occupation and methylation profile analyses of CTCF-binding site, followed by stepwise screening and verification using MS-HRM and mass spectrometry analysis, this study identified a set of colorectal neoplasia-specific methylation biomarkers with robust performance in diverse sample sets. The top five biomarkers, named as CTCF_13, CTCF_33, CTCF_55, CTCF_94, and CTCF_113, were selected and recommended as biomarkers for early detection of colorectal neoplasia. Compared to the performance of two currently acknowledged robust methylation biomarkers (*BMP3* and *NDRG4*) in the same sample set, all of the five new markers exhibited a more robust discriminatory performance. In addition, when the five new biomarkers were considered as a marker panel and tumor-positive was defined as having two or more (of the five) positive biomarkers, the discriminatory performance improved significantly compared to that by the best single marker.

Although many biomarkers based on DNA methylation of gene promoters have been proposed as useful early detection markers for CRC [2, 4–14], the accuracy of these biomarkers still needs to be improved, particularly with regard to the detection accuracy of adenomas. Currently, the most established methylated DNA blood biomarker is methylated *SEPT*9. *SEPT9* is now commercially offered as a blood-based screening test in various assays. Furthermore, the sensitivity and specificity of the *SEPT9* assay for CRC was comparable with that of the conventional FOBT, confirming its potential usefulness as a blood-based biomarker for CRC [16]. However, a recent study demonstrated that the methylated SEPT9 assay was superior to FIT at detecting CRC neoplasms, but both approaches were suboptimal for diagnosing patients with advanced adenomas [17], which underscored the need for further improvement of this test for implementation of a population-based screening of colorectal neoplasia. In addition, although the sDNA test has culminated in the development of an FDA approved, clinically available stool-based CRC screening test, its sensitivity for detecting advanced precancerous lesions is only 42.4% [13]. In summary, although impressive accuracy for CRC detection has been achieved based on DNA methylation biomarkers, both serum-based DNA test and stool-based DNA test were less sensitive for CRC precursor lesions, particularly for adenomas [4-6, 13]. However, this study identified a set of colorectal neoplasia-specific methylation biomarkers, viz., CTCF_33, CTCF_55, CTCF_94, CTCF_113, and CTCF_13, with high accuracy both for colorectal adenoma and CRC. Notably, as the only marker whose methylation level in tumor tissues was significantly lower than that in normal tissues, CTCF_33 was more sensitive for adenoma than for CRC (Table 3). In particular, when the five new biomarkers were considered as a marker panel and tumor-positive was defined as having two or more (of the five) positive biomarkers, the marker panel could achieve a sensitivity of 91.67% for adenomas with a specificity of 94.05%. In other words, compared with the methylated SEPT9 assay and the sDNA test, our new marker panel exhibited significantly higher accuracy for adenoma and afforded sensitivity and specificity of >90% both for adenoma and CRC (Table 5). In addition, although the sDNA test may be more accurate than the methylated SEPT9 assay in detecting colorectal neoplasia [5], it includes quantitative molecular assays for *KRAS* mutations, aberrant *NDRG4* and *BMP3* methylation, and a hemoglobin immunoassay. By comparison, our new test only required detecting the aberrant methylation of five CTCF-binding sites, indicating that it is more labor- and cost-effective, which is very important for the implementation of this test for population-based screening of colorectal neoplasia.

Furthermore, compared with the existing biomarkers that are based on DNA methylation in the promoter region, biomarkers based on the methylation state of CTCF-binding sites showed many other advantages: (1) CTCF-binding sites are widespread in human genome and most of their locations on human chromosome are already known, facilitating genome-wide screening and validation of biomarkers for tumor prevention and treatment. (2) Compared with CpG islands in the promoter region, which are approximately 200 bp in length, CTCF-binding sites only have a sequence length of around 30 bp [46], which is more conducive to the development of DNA methylation biomarkers with high detection sensitivity and accuracy. This is particularly true for serum- and stool-based DNA tests for the early detection of CRC because tumor DNA content in blood or stool samples is very low and is degraded, and tumor DNA is further degraded after bisulfite treatment. In this situation, methylation biomarkers with shorter target sequences will be more advantageous. (3) Aberrant DNA methylation of CTCF-binding sites has been connected for all types of tumors; therefore, using a strategy similar to that described in our study, CTCF-binding sites could be potentially developed into biomarkers for the prevention and treatment of other tumors.

To the best of our knowledge, this is the first study for colorectal neoplasia-specific methylation biomarkers based on CTCF DNA-binding sites in a screening setting. Moreover, these biomarkers may be useful in tumor prevention and treatment with respect to factors such as early detection, diagnosis, tendency of tissue invasion, metastasis, prognosis, and response to chemotherapy. Using a strategy similar to that of this study, CTCF DNA-binding sites could be potentially developed into biomarkers for other tumors. In summary, this study presents a novel strategy to develop biomarkers for tumor prevention and treatment based on CTCF DNA-binding sites. We hope that our study will serve as a starting point for more extensive and in-depth studies to fully estimate the potential of CTCF DNA-binding sites as methylation biomarkers for clinical applications in tumor prevention and treatment.

## MATERIALS AND METHODS

### Tissue samples

Two hundred and ninety-five fresh colorectal tumor tissues and eighty-four matched normal tissues were obtained from surgical resections, fresh-frozen, and stored at −80°C. Samples of normal tissue were obtained from a distance of at least 6 cm from the tumor and were confirmed to be tumor-free by microscopy. These patients had undergone curative surgery at the First Affiliated Hospital of Kunming Medical University between 2014 and 2016. Patients gave informed consent in writing to use their bowel tissue for research. The selection was solely based on the availability of tissues for the study, and we did not exclude patients with a family history of CRCs. Clinicopathological information, including age, sex, tumor location, and tumor stage, were obtained from these patients (Supplementary Table 2). Tumors were staged on the basis of the pathological tumor-node-metastasis (pTNM) staging system of the American Joint Committee on Cancer (AJCC). The study was approved by the First Affiliated Hospital of Kunming Medical University Ethics Committee.

### Candidate CTCF-binding site discovery

Wang *et al.* analyzed genome-wide occupancy patterns of CTCF in 19 diverse human cell types, including seven immortal cell lines and 12 normal cell types [29]. They identified 4146 specific CTCF-binding sites whose occupancy was distinguished in immortal cell lines, epithelia, fibroblasts, and endothelia. Among the 4146 CTCF-binding sites, the occupancy of 1236 sites are specific only in immortal cell lines, and these binding sites can distinguish immortal cell lines from normal cell lines. By analyzing the association data between genome-wide occupancy and the methylation state of these 1236 CTCF-binding sites in 13 cell lines (including six immortal cell lines and seven normal cell lines) published by Wang et al. [29], we identified 121 CTCF-binding sites whose occupancy was significantly associated with methylation. At these 121 sites, increased methylation was negatively associated with occupancy, indicating that the CTCF occupancy at these 121 sites is specific in cancer cells and is significantly associated with methylation. In theory, the DNA methylation status of these sites can be used to distinguish cancer cells from normal cells. We hypothesize that these 121 CTCF-binding sites can be used as candidate biomarkers to distinguish tumor tissues from normal tissues.

### DNA extraction and bisulfite modification

Whole genome DNA was extracted using the QIAamp DNA Mini Kit (QIAGEN, Germantown, MD). Bisulfite modification was performed using an EpiTect Fast DNA Bisulfite Kit (QIAGEN, Germantown, MD) according to the manufacturer’s instructions.

### MS-HRM analysis

MS-HRM analysis is based on the comparison of the melting profiles of PCR products from unknown samples with profiles specific for PCR products derived from methylated and unmethylated control DNAs. The protocol consists of PCR amplification of bisulfite-modified DNA with primers designed to proportionally amplify both methylated and unmethylated templates and subsequent HRM analysis of the PCR product. Because it is a labor- and cost-efficient, high-throughput, and sensitive technology for single-locus methylation detection [38], we performed first screening of these 121 candidate CTCF-binding sites against 33 pairs of normal and tumor tissues using the MS-HRM approach to eliminate markers that did not show evidence of tumor-associated methylation. The thirty-three tumor tissues tested included six adenomas, nine Stage I CRC, 10 Stage II CRC, and eight Stage III CRC tissues. MS-HRM PCR primers were designed for 121 candidate CTCF-binding sites according to their genomic DNA sequence [29] and their most possible binding positions [27]. The most possible CTCF-binding positions for each CTCF-binding site was predicted by using the online database CTCFBSDB 2.0 (http://insulatordb.uthsc.edu/), which now contains almost 15 million CTCF-binding sequences in 10 species [27]. MS-HRM PCR product for each CTCF-binding site must include the most possible CTCF-binding positions. We followed the MS-HRM primer design guidelines as previously outlined [38]. MS-HRM primers and chromosomal locations for 121 candidate CTCF-binding sites are listed in Supplementary Table 3. PCR amplification and HRM analysis were performed on an ABI StepOne Plus real-time PCR system (Applied Biosystems, USA) using MeltDoctor™ HRM Master Mix (Applied Biosystems, USA) according to the manufacturer’s instructions.

A standard curve with known methylation ratios was included in each assay and was used to deduce the methylation ratio of each tumor and normal tissue. The HRM data were analyzed using the ABI high-resolution melting software (Applied Biosystems, USA). Output plots are in the form of normalized melting curves and difference plots.

### Quantitative high-throughput DNA methylation analysis by mass spectrometry

Because MS-HRM only provides a semiquantitative measure of cytosines that have been converted to thymines and is only used for qualitative comparisons and not for quantitative analysis [38, 39], mass spectrometry was used to further screen and validate the CTCF-binding sites identified by MS-HRM with the SEQUENOM MassARRAY platform (Agena Bioscience Inc., San Diego, CA previously Sequenom Inc.) using the EpiTYPER^®^ T Complete Reagent Kit as previously described [40, 41]. By comparing the performance of all widely used DNA methylation analysis methods that are compatible with routine clinical use, MassARRAY analysis has been confirmed to be a reliable method when validating DNA methylation differences in large cohorts and is also an excellent technology for developing epigenetic biomarkers [39]. MassARRAY is an accurate, sensitive, and reproducible high-throughput quantification technology for DNA methylation profile that is compatible with automation [40, 42]. Bisulfite-treated DNA was subjected to PCR and then processed following the manufacturer’s instructions. The methylation status of a detected pattern was then analyzed using Epityper software version 1.2 (Agena Bioscience Inc., San Diego, CA, USA). PCR primers were designed by using the online tool EpiDesigner (www.EpiDesigner.com) (Agena Bioscience Inc., San Diego, CA, USA) and are listed in Supplementary Table 4. After the T-cleavage reaction, the resulting fragments of each CTCF-binding site may contain more than one CpG dinucleotide, and are, therefore, referred to as “CpG units.” The CpG units that produced data for less than 30% of samples (unreliable CpG units) and samples lacking more than 30% of their data points (unreliable samples) were discarded [43]. The methylation status of each CTCF-binding site was represented by the CpG unit with the highest area under the curve (AUC) value according to a receiver operating characteristic (ROC) analysis.

### Statistical methods

Hierarchical clustering analysis of the MassARRAY data was performed using the average method via R package pheatmap [44]. The distance matrix used for clustering was calculated using the R package vegan [45].

AUC values were estimated using MedCalc software (version 9.2.0.0; Broekstraat, Mariakerke, Belgium). Sensitivity and specificity were estimated by 1000 bootstrapping runs. We report sensitivity at the 90% or 95% specificity level from the 1000 bootstrap runs at 95% confidence intervals. *P* < 0.05 was considered statistically significant. For the five-marker panel analysis, we considered two or more markers positive as tumor positive.

## SUPPLEMENTARY MATERIALS TABLES








