# Supplementary material for: Identification and validation of colorectal neoplasia-specific methylation biomarkers based on CTCF-binding sites
Source: Oncotarget. 2017 Dec 11;8(69):114183–94. doi: 10.18632/oncotarget.23172 (PMC5768395; doi:10.18632/oncotarget.23172)
Supplement: Supplementary file 1 [file oncotarget-08-114183-s001.pdf]

Identification and validation of colorectal neoplasia-specific methylation biomarkers based on CTCF-binding sites

SUPPLEMENTARY MATERIALS

Supplementary Table 1: Tumor specificity of the 23 candidate CTCF-binding sites identified by MS-HRM analysis

See Supplementary File 1

Supplementary Table 2: Sample informations used in this study

	Normal Tissues(n=84)	Tumour tissues				
		Adenoma (n=108)	Stage I (n=39)	Stage II (n=101)	Stage III (n=47)	Total (n=295)
Male, n (%)	48 (57%)	65 (60%)	20 (51%)	62 (61%)	24 (51%)	171 (58%)
Female, n (%)	36 (43%)	43 (40%)	19 (49%)	39 (39%)	23 (49%)	124 (42%)
Age, median (range)	57 (24-81)	59 (20-86)	58 (37-81)	57 (28-80)	55 (36-82)	58 (20-86)
Colon, n (%)	42 (50%)	76 (70%)	9 (23%)	54 (53%)	19 (40%)	158 (54%)
Rectum, n (%)	42 (50%)	32 (30%)	30 (77%)	47 (47%)	28 (60%)	137 (46%)

Supplementary Table 3: MS-HRM primers and chromosoma locations of the 121 candidate CTCF-binding sites

See Supplementary File 2

Supplementary Table 4: Primers designed for mass spectrometry analyses

See Supplementary File 3
