# Supplementary material for: Identification and validation of colorectal neoplasia-specific methylation biomarkers based on CTCF-binding sites
Source: Oncotarget. 2017 Dec 11;8(69):114183–94. doi: 10.18632/oncotarget.23172 (PMC5768395; doi:10.18632/oncotarget.23172)
Supplement: Supplementary file 2 [file oncotarget-08-114183-s002.docx]

**Supplementary Table 1**

Tumor specificity of 23 candidate CTCF-binding sites identified by MS-HRM analyses

“n” represents that it is not tumor specific with this CTCF-binding site in this sample

“T” represents tumor tissue and “N” represents normal tissue
