# Supplementary material for: Identification and validation of colorectal neoplasia-specific methylation biomarkers based on CTCF-binding sites
Source: Oncotarget. 2017 Dec 11;8(69):114183–94. doi: 10.18632/oncotarget.23172 (PMC5768395; doi:10.18632/oncotarget.23172)
Supplement: Supplementary file 3 [file oncotarget-08-114183-s003.docx]

**Supplementary Table 3**

MS-HRM primers and chromosoma locations for 121 candidate CTCF-binding sites

| CTCF Site | Forward primer sequence (5'to3') | Reverse primer sequence (5'to3') | chrom | chromStart | chromEnd |
| --- | --- | --- | --- | --- | --- |
| CTCF_1 | GATAGTGGTGGTGGAGGGTAGT | CGTATTAATCCCTCCTCCTACAA | chr1 | 1297502 | 1297609 |
| CTCF_2 | tGGTttGCGTTTTTTtGGGT | GCTCaCCTACGCCCCaAAT | chr1 | 27286806 | 27286916 |
| CTCF_3 | CGTGATGAGTAAAGTGGAGAAGG | ACATCTCTCTCCGCTATCACCTA | chr1 | 31158052 | 31158150 |
| CTCF_4 | AAGTttGGGGGAGAGGtAGA | TCaaAACaAaACTTAaCTCaCCTA | chr1 | 43751296 | 43751418 |
| CTCF_5 | TATCGTGCGGGTAGGGGTT | AACTCCCACTTACGCCCCA | chr1 | 116711116 | 116711246 |
| CTCF_6 | GGTTTAGTAGGAAGACGTTTAGAGG | ACAAACCTACAAACCCTTTACACA | chr1 | 163039125 | 163039284 |
| CTCF_7 | TTATTTGATTAAGGAAAAGGAAGGT | GAACCGCTCAATCTCTATCTCC | chr1 | 183155374 | 183155478 |
| CTCF_8 | ATTGGGGTTTTTTATATTAGGTGTT | CGAAATTACTACCAAATCCCAA | chr1 | 185287255 | 185287368 |
| CTCF_9 | GTTGGGAGAGTCGGTATTGG | CCGAAAAAACAACTACAACCAA | chr1 | 201278767 | 201278863 |
| CTCF_10 | CGGGAATTGATTGGTGGGT | CAACAAAAAATTTAACCCCACCTAC | chr1 | 224828922 | 224829011 |
| CTCF_11 | TTCGCGGTCGAGGAGGATT | TTACaCCCGATTTCTCTCCaaCC | chr1 | 226099140 | 226099245 |
| CTCF_12 | TGAGTTGTGGAATGAATAGGGTGT | AACGCAAACTCTACACACCCTTC | chr10 | 1758054 | 1758177 |
| CTCF_13 | ATTTAGCGTTTTAGTTATTAGTTGGGT | CCCAAAAACTCTAACCCTCCTC | chr10 | 15761963 | 15762083 |
| CTCF_14 | TAGTAGCGGTAGTGAAAGGGAA | AACCACTTACAAACAACCTCTACC | chr10 | 34594238 | 34594308 |
| CTCF_15 | GGTAGTTGGAGGGTTGTAGGTT | CGCAATATACGACGAACCCTA | chr10 | 88392058 | 88392190 |
| CTCF_16 | TTTGAATTTGAATTTGTTGAAGAAGT | AAATAACTCCAACCTCTCCCTCTA | chr10 | 94180015 | 94180097 |
| CTCF_17 | tGGAGGCGtCGtAGATtGAGAT | GCGCTCCCaCaAATCCTAAC | chr10 | 94822465 | 94822603 |
| CTCF_18 | AAAGGTAGAAGTGGTTAATGGGTT | CCAACTCTACAACTCACCTACTACAA | chr10 | 102807874 | 102807969 |
| CTCF_19 | TTGGGATTTATCGGAAGGAGAG | CCGTAACGCCCCCTAACC | chr10 | 115999244 | 115999339 |
| CTCF_20 | TTTTAGAGTAGCGAGTTGGAGG | CTAAAACCTTTACTCGACTACTCCC | chr11 | 63374083 | 63374185 |
| CTCF_21 | TGGAAGGGATAGGGTGGGT | AACGAAACGACCCTAAACCTAA | chr11 | 63768010 | 63768089 |
| CTCF_22 | GCGTTTTGTTTGTTTTTAGGGT | ACAAACTAAATCCCTATCCCTACC | chr11 | 68611478 | 68611587 |
| CTCF_23 | GACGGTGAGTGTTGTGAATAGGT | CTCCCTCTACTATCACCACTAAACC | chr11 | 134263569 | 134263649 |
| CTCF_24 | ATGTGAAGGCGTTGAGGGTT | CTTACTTAAAACTCCGAACACCAAC | chr12 | 2566810 | 2566877 |
| CTCF_25 | TTGTTGTAGGAGGAAGAGGTTGG | CGCCTTCCATCCCTACTCCTA | chr12 | 6665154 | 6665250 |
| CTCF_26 | TAGGGGCGATGGAGAGTAGG | AAACACGCCTTTTTAACTCTAACC | chr12 | 39539382 | 39539498 |
| CTCF_27 | GGGATTCGGGTATTTAGGGT | CGTAAACCCAAAAAACCAACAA | chr12 | 54785180 | 54785276 |
| CTCF_28 | TCGGTAGGAAGGTTTAGTATTGGT | CTACCCAACAATTCACATAACCTC | chr12 | 67788561 | 67788646 |
| CTCF_29 | GAGCGGAGGAAGGATAGAAAAG | CAAACCCTAAAACGACAAAACAA | chr12 | 122356321 | 122356447 |
| CTCF_30 | AACGAGGTtGGATtTtGGTTAGT | aAACCCTAACTACCaAAACCTaCTCTA | chr12 | 124854047 | 124854195 |
| CTCF_31 | CGTTTAGTTTTTAGAATTGGAAGAGA | TTTCTTAAAATCCGAATACACCAA | chr12 | 124864543 | 124864670 |
| CTCF_32 | CGTTGGTTTAGTTTTTAAGAGGGAT | GCCTCTCACACAAATTCAACTCC | chr12 | 126168444 | 126168522 |
| CTCF_33 | TTGAGGTTAGGGTTTTTATTTAGAGT | CCTCTTCCACTTAACCTATCTTCAA | chr13 | 61564369 | 61564467 |
| CTCF_34 | GAGGAGTTTTAGCGTTTGTTAGG | AACGAAAAACCGACACTACCA | chr14 | 37116418 | 37116512 |
| CTCF_35 | TTTGTCGTGGAGAGGGAAAGT | TCGACTCTAAAAACCTAAATCCCTA | chr14 | 38066939 | 38067056 |
| CTCF_36 | TAGGTTATAGGAGAGGGTTATGGAT | AAATACAAATCCTCCCAACTCC | chr14 | 75040173 | 75040279 |
| CTCF_37 | CGTAGGtGGtGTTAGTtGGGtT | CCGAaACCGATAaaTTACCCTC | chr14 | 104808428 | 104808544 |
| CTCF_38 | TTGGTGTTGTAGTAGTTTTGGTTGT | CCGCTATACGAAAAACCAAAC | chr15 | 22387584 | 22387716 |
| CTCF_39 | TTCGGGACGTtGTtGGAGG | AACCGaATCTCCTAAaaACCCaTAC | chr15 | 67326057 | 67326161 |
| CTCF_40 | GGtGTCGTtGATCGTTAGtGGATT | aACaACGaAACCGCCaAACC | chr16 | 2140904 | 2141019 |
| CTCF_41 | GTAGTtGTAGCGAtGGGTAGGAT | CaCaCCGCaCaACTCaACaC | chr16 | 7136988 | 7137086 |
| CTCF_42 | TCGGGAGtTTtTTAGtGTtGtGG | CaAATAaACCGAACGAACCCaA | chr16 | 28935920 | 28936068 |
| CTCF_43 | GAtGCGTtTAGGAAATAAGGAGG | CCTAaaTACCCCCTAATATCCTCaA | chr16 | 33619149 | 33619236 |
| CTCF_44 | GACGCGGAtGTAGtttAGGAAG | AAaTaACGAAAATCTCCTCTaAATCC | chr16 | 54971435 | 54971528 |
| CTCF_45 | TCGACGGAAtTtGAGGGAtT | aaACCCTAACCTAACCCaAAACC | chr16 | 55357784 | 55357901 |
| CTCF_46 | TTCGTATTtGtGTAGAGtGGTtGT | AAaCCCCCTTTTCaCCaCTA | chr16 | 68273990 | 68274119 |
| CTCF_47 | tTAGGAATAGtCGtGTtGGtTtGT | ACCTAaTATCCGCaCaCCCTT | chr16 | 85221191 | 85221264 |
| CTCF_48 | TtGAGTAtGGGAAGTtTtGAAAGG | CGACTCCCTATaACTACCCTAACCTA | chr16 | 88825715 | 88825811 |
| CTCF_49 | GGTtTTAGGTtGGTATtGTtGGGT | CCTAATCCaACaTACTCaAAATCCaA | chr16 | 88953353 | 88953473 |
| CTCF_50 | TTCGGGGTAGtTAGGAGGGT | AATaCaCTAAACGAAAaCTTCCTCaT | chr16 | 89002551 | 89002636 |
| CTCF_51 | GTTTtGTTAGTTTCGtGGGGTT | ACaACaCaAACaACCTCGACC | chr16 | 89641132 | 89641253 |
| CTCF_52 | tGTtGtGTtGAtttTAGCGAGGGT | CGTCCTACCCaCaTCaCaaAACTC | chr17 | 22012579 | 22012691 |
| CTCF_53 | GGAtTtGttGGAGTTAGtGGGT | ACGCTCTAaTTTCCTCaTCCTC | chr17 | 25886712 | 25886842 |
| CTCF_54 | GtCGGAAGCGGAtGtAtGtGAT | CTAATCaACCCCCGACTACaACC | chr17 | 27893309 | 27893384 |
| CTCF_55 | GAGTtGGAGCGTAtTTtGAGGT | CGAAATTTATCTAACTCCTAACCCT | chr17 | 27940559 | 27940669 |
| CTCF_56 | ttGCGTAATtGTtGGAGTtGGT | ACCGACTACaAaTACaACTTCaCCTA | chr17 | 36715828 | 36715909 |
| CTCF_57 | CGGtTTCGATTAGGtGAtGGAT | GCTCaCCTTCaCCaCCTCCTC | chr17 | 40700271 | 40700397 |
| CTCF_58 | GGAACGGTtGGGAGTAGGAAGT | CTTTACaACCaCCCGAaACCTCTA | chr17 | 64733726 | 64733841 |
| CTCF_59 | tGATtGCGtGTCGGGTATtGT | CaCGTCaAaaaACGaaTATACCTCaC | chr17 | 75143130 | 75143206 |
| CTCF_60 | CGAGGGtGTAGAAGGTTAGAGGtG | CCGACTACTCCaCaATACTCaCaAC | chr17 | 79438878 | 79439002 |
| CTCF_61 | GATtTtGGATAAGGATAAGGAAGGT | CTAACCaTaCCTAaAaaCaTaCCTCC | chr18 | 9722789 | 9722870 |
| CTCF_62 | GTTTCGGtGGGtGATtGtGGT | CaACaCTAACCAAaCaCCTTCaaCC | chr18 | 47794937 | 47795033 |
| CTCF_63 | TtGtCGGTGTtGGAGTAtTTtGAG | CaAaCGCGCCCaCaTCaC | chr18 | 77139078 | 77139191 |
| CTCF_64 | CGGGATTtGGtTTTTtGGGT | GCCTCCTCaACGaACaAaCCTAC | chr19 | 2425391 | 2425526 |
| CTCF_65 | GtTtTCGGAGtTTTtGGGTtGT | CCGTATTCTTCTACaaCCTCaTAAaC | chr19 | 3783750 | 3783846 |
| CTCF_66 | ATCGGGGAGTtTtGGGAAT | CaACTAAACTCGCCaaCCTCC | chr19 | 7661591 | 7661695 |
| CTCF_67 | TCGGAAtAAAATtGtGTTtGGGT | TTCaATAaCaaCaCCCCaCCTaaC | chr19 | 15870903 | 15871018 |
| CTCF_68 | GTTTAGGGGttGGAGtGAGAtttGT | CaAAACTCCCGAACTCaAACaACTA | chr19 | 34242958 | 34243069 |
| CTCF_69 | AAGGAGGTTTAGttGAtTttGGGTT | ACGaAACCCGACTAACCTCCaC | chr19 | 46915289 | 46915445 |
| CTCF_70 | GAGGtTtAAtGTTTTAGGAAGAGGT | TCCCaCTAaCTCTaCCCCaaCTA | chr2 | 21618400 | 21618531 |
| CTCF_71 | ATTTAAGGtTCGGtttGGAGAGG | GCCTCaACTCCCCaAAACaA | chr2 | 66672422 | 66672520 |
| CTCF_72 | GTTtTTtTAtGCGTAGGtGTtTAGG | CTAATTTaAAAaCCCaCTACCCaaA | chr2 | 121321765 | 121321914 |
| CTCF_73 | GtGATtTtGTTTtTtGAGTtGTAGGT | AaCCTaCCCCaCaATCaACaaTC | chr2 | 238509070 | 238509217 |
| CTCF_74 | GGATAGtTttCGTtGGAGGTAGTT | CCaaaCTCTCCCaCCaACaTaC | chr20 | 22564121 | 22564250 |
| CTCF_75 | tCGTAGAGGtCGttGTAGGATtG | TACaaTACGaTCCCTAAAACTCCC | chr20 | 23029264 | 23029376 |
| CTCF_76 | tGTtTtGGAAtGTtTTTtGGAGGT | CCGaaTACTACTAAAAACCTTaCCCa | chr20 | 56522931 | 56523023 |
| CTCF_77 | GGAAGGGAtGGGGAATAGGT | CGaACCaCCaCaCCCAACTAC | chr20 | 58319247 | 58319355 |
| CTCF_78 | AAtGAAtGAAttGGGAAATAGtGT | TTCCaTCaaTTCaACaTATaTCaaC | chr21 | 27302623 | 27302734 |
| CTCF_79 | CGAtGGAGGTTAGGGtTAGTAGGt | CGCTCCaTCTCCCGAACC | chr21 | 28337819 | 28337930 |
| CTCF_80 | GAttTtTCGTAGGtGTtGTtGGGT | CCaACGAAaACCaAATCaAAACC | chr21 | 44866623 | 44866705 |
| CTCF_81 | GGtGAGTtCGtGGGGTAGTttGT | TCaCTACaCaACTCaACaACaCaTCC | chr22 | 24503762 | 24503859 |
| CTCF_82 | GtTAtTTAGAAGGtGtTtGtAGGAGG | CGaCTaCCGATaAACACCCaaAC | chr22 | 38214854 | 38214933 |
| CTCF_83 | CGAtGTCGTAGtGTtGTAGGtGTAG | ACGaCaCCCaACTCCTAAaCCTA | chr22 | 41633692 | 41633802 |
| CTCF_84 | TAGGCGAGGAGttttGTAAAGtGT | aCCCaAAAACGATCCCaACaAA | chr3 | 13324736 | 13324817 |
| CTCF_85 | GAATtGGGCGGGAAGGAG | TACCGCaaCGAAAACaCCTACaC | chr3 | 48693627 | 48693748 |
| CTCF_86 | GTtTtCGTACGCGGtGTAGGTtG | ACGACTACGCCGTAACCaAACC | chr3 | 128722756 | 128722844 |
| CTCF_87 | GTTCGGGtGAGGTAtTtGGAGG | CGAAACCGTCTCCCTCCaAA | chr3 | 129062877 | 129062982 |
| CTCF_88 | AGGAAtTtAGGGtGGAAGAGAGAA | AaCaTAACaaAACaCCaCCTCaCTaA | chr3 | 181666269 | 181666384 |
| CTCF_89 | GTTTCGTTtGTtTtTtGtGGTAGT | CCGAaCTCaAACCaACCCT | chr4 | 1026409 | 1026503 |
| CTCF_90 | GGATATTtGTTtGTtGGTAtTtGAGT | TACCaCaACaCaCCCTACaCaCC | chr4 | 1606733 | 1606837 |
| CTCF_91 | TCGTtGGGGTtGTAGtGTtGAGT | CCGACaATTCCaaATCCTCCa | chr4 | 8276772 | 8276833 |
| CTCF_92 | GGAGGTtCGGtGTtAGGGA | CGCaACCCaACCCaCTTA | chr4 | 56502373 | 56502521 |
| CTCF_93 | TtTTCGGGGAGGTAGTAGTtGT | CGAAaaTTTCCTCTTTCCTTTAC | chr4 | 81123441 | 81123582 |
| CTCF_94 | GAGAGAAGGGGTtTTTAGtGAGGT | GCTCCaCCCGCaaCTAaCaa | chr4 | 165304966 | 165305120 |
| CTCF_95 | GtGGATttGGtTAGTTAGTtGTtGT | ACTCaATATaTCTAAACaACCCCTTC | chr4 | 186577840 | 186577937 |
| CTCF_96 | ATTAGTAGGtGGAGATAGGAtGAGG | CCaaCaATAaCCaCaACaAACC | chr5 | 17883292 | 17883368 |
| CTCF_97 | ATACGGTtTAGACGGtttTtGGtGT | CGACaCCTATAAATCCCCaAACC | chr5 | 37837555 | 37837693 |
| CTCF_98 | GGTCGttCGGGTtCGGtG | ACCGACGCaCaCTCTACaACaA | chr5 | 176023847 | 176023940 |
| CTCF_99 | AAtGAtTTTtAGGGtTttGGtGGT | CCaACGaaTCCGAaTCTCCTA | chr6 | 78172164 | 78172288 |
| CTCF_100 | AAtAAAAGAtGAGTtTtGGAAAGGT | CGaAACTTACaAaaCCTCaACTCC | chr7 | 1952600 | 1952680 |
| CTCF_101 | GTtAtGAAGttTAGtGGTtGGGtGT | CTAAAaaCGACCGTaaCCCTAAATC | chr7 | 26660172 | 26660290 |
| CTCF_102 | GGGATtAGGTtGGGTTtTtAGGT | CGCCaaaCTCaACCaCCaCa | chr7 | 127910964 | 127911065 |
| CTCF_103 | tCGtAGAAGAGCGGGAAGGT | CGCTACTAACTCTAACCCTCTTACC | chr7 | 127992351 | 127992468 |
| CTCF_104 | ATCGGGTtCGAAGtGTAtGGT | GTAAaaTCaAACTCCCTTCCCTC | chr7 | 143317292 | 143317432 |
| CTCF_105 | CGGAGGTTAGGAGGGGTtGTAG | TAAACGCCAACGAATCCTCCa | chr7 | 150706516 | 150706631 |
| CTCF_106 | TtGGGttGAtAATAGAGAGATtGGT | GaAaCCTTCTCaCCTTAAACaACC | chr7 | 153078761 | 153078881 |
| CTCF_107 | TCGGGTAtCGTtTtGGAtttGT | ACTCCaaCaAaACCTATTCTTCCaA | chr7 | 154650073 | 154650180 |
| CTCF_108 | CGGTTtTTtGGGAAGTAGAAGAAT | TAACGAAATCCCGACaAaACaAC | chr7 | 155867807 | 155867903 |
| CTCF_109 | tGAAGTAGAGAGGGAGAGGAGAGAT | CaTTAaACaaCGTACaCCTTACCTTC | chr8 | 702239 | 702330 |
| CTCF_110 | GTATTtGCGTTAAGAAGGTTtGGAT | CCaAAAaTCaACaAAATCaCaCTCC | chr8 | 1878957 | 1879071 |
| CTCF_111 | AGAGTCGGAGTtGAGTTAGGGAGT | CaCGTAAACTAaACTCaAaAACCCT | chr8 | 27441256 | 27441376 |
| CTCF_112 | TTAGTAtGtGGtGttAGttGGTtTtG | ACCaaAaCCGAaACCTaAaTACC | chr8 | 27761371 | 27761456 |
| CTCF_113 | GGGAAAttCGAGGTAGAGGttT | TACGCCCCTCGaCaCaACT | chr8 | 69243102 | 69243188 |
| CTCF_114 | tGtTtGAGTAGGtGAGAGTAGGtAA | CaCaACCaATAaaCaAAACTACCTA | chr8 | 139926781 | 139926862 |
| CTCF_115 | TtGCGtTTTtGGCGGGAttT | GCCGACTACCCaACaACCTAC | chr8 | 145750200 | 145750298 |
| CTCF_116 | tGTTtAGATACGtTtGAGAAGTAGG | CaTACCGTTTCCTTTCTACaACC | chr9 | 37921686 | 37921748 |
| CTCF_117 | GGtTATAAGGtGAGtAGTTAGGTAGG | CCTATCATCCTTTCCTCCaaAC | chr9 | 97807392 | 97807502 |
| CTCF_118 | GtAGAGAGGAGGAATtGGTAttGAA | CCaCGCTTTTCTTCCCaAA | chr9 | 100069452 | 100069558 |
| CTCF_119 | GATAGGGTAGTTTTAGGTtTtGGTT | CCaATCTCCTCTATCTATCaAATCC | chr9 | 129425068 | 129425185 |
| CTCF_120 | AGGGTTAGTCGGAGtGGTTTAG | aAAACaACaACTCCCaaCTTCC | chr9 | 137417843 | 137417965 |
| CTCF_121 | TAGCGTCGTCGAtGtGGGAG | CCGCCaCCGCCTCTAAATAC | chr9 | 137483833 | 137483952 |

Note: chromosoma locations were mapped to the human genome (GRCh37/hg19)
