# Supplementary material for: Identification and validation of colorectal neoplasia-specific methylation biomarkers based on CTCF-binding sites
Source: Oncotarget. 2017 Dec 11;8(69):114183–94. doi: 10.18632/oncotarget.23172 (PMC5768395; doi:10.18632/oncotarget.23172)
Supplement: Supplementary file 4 [file oncotarget-08-114183-s004.docx]

**Supplementary Table 4**

Primers used for mass spectrometry analyses

| Target Name | Primer Name | Sequence (5'to3') |
| --- | --- | --- |
| CTCF_13 | CTCF_13-C-502-10F | aggaagagagGATTGTTTTTGAGATGGGATTGTAA |
|  | CTCF_13-C-681-T7R | cagtaatacgactcactatagggagaaggctAAAACTAACCCAAAAACTCTAACCC |
| CTCF_14 | CTCF_14-471-10F | aggaagagagATTTTAGGTTTTAGGTATGGGTTGG |
|  | CTCF_14-644-T7R | cagtaatacgactcactatagggagaaggctCAAATAAACAAATAAAAAACCTCCC |
| CTCF_33 | CTCF_33-570-10F | aggaagagagTTGAGGTTAGGGTTTTTATTTAGAGT |
|  | CTCF_33-725-T7R | cagtaatacgactcactatagggagaaggctCTAaaTCTACTATCaTTCTaCCaACC |
| CTCF_35 | CTCF_35-C-460-10F | aggaagagagGTTATTGAGATGGAGAGAGGTTGG |
|  | CTCF_35-C-702-T7R | cagtaatacgactcactatagggagaaggctAAACTTAATCCAAATCAAAAATCCC |
| CTCF_39 | CTCF_39-C-425-10F | aggaagagagTTGGAAGTTTATGTAGAGGAAGTGG |
|  | CTCF_39-C-634-T7R | cagtaatacgactcactatagggagaaggctAAAAACCCTAACAAAAAACAAAACC |
| CTCF_41 | CTCF_41-473-10F | aggaagagagTGGTTGAGAGTTTTGTATAGAGATGG |
|  | CTCF_41-653-T7R | cagtaatacgactcactatagggagaaggctTAAAAACAACATCCAATCAAATCAA |
| CTCF_47 | CTCF_47-290-10F | aggaagagagGTAGTGTTGGGGGTAGTTTTAGGTT |
|  | CTCF_47-634-T7R | cagtaatacgactcactatagggagaaggctCCACCCAAATAAAAACAAATAAAAA |
| CTCF_48 | CTCF_29398-536-10F | aggaagagagTTGAGTATGGGAAGTTTTGAAAGG |
|  | CTCF_29398-630-T7R | cagtaatacgactcactatagggagaaggctACTCCCTATaACTACCCTAACCTA |
| CTCF_50 | CTCF_50-C-641-10F | aggaagagagGGAGATTAGGATGGGAGATATTTG |
|  | CTCF_50-C-314-T7R | cagtaatacgactcactatagggagaaggctATCCAAATAAAAACTCCAAAAAACC |
| CTCF_55 | CTCF_55-C-394-10F | aggaagagagTTGTTTGGTTTTTGGTTTTGG |
|  | CTCF_55-C-625-T7R | cagtaatacgactcactatagggagaaggctACCCAACTCAAAACCAAATTCC |
| CTCF_60 | CTCF_60-414-10F | aggaagagagGGTTTTGGGTAAAGTATTTTTGGTT |
|  | CTCF_60-654-T7R | cagtaatacgactcactatagggagaaggctTACCTACTAATCCTTCAAAATCCCC |
| CTCF_69 | CTCF_69-C-770-10F | aggaagagagAATTTGTTAGTGTTTATGTTTGAGGA |
|  | CTCF_69-C-475-T7R | cagtaatacgactcactatagggagaaggctACCCAATTAATCTTAAACCTCCATC |
| CTCF_71 | CTCF_71-C-405-10F | aggaagagagAGGTGGAGAGGTTATTTTTTTTGTT |
|  | CTCF_71-C-674-T7R | cagtaatacgactcactatagggagaaggctAAACTCACCCAAATTTCAACTAATC |
| CTCF_75 | CTCF_75-C-442-10F | aggaagagagGTTTAGGGGTATTGGGTTAGGG |
|  | CTCF_75-C-751-T7R | cagtaatacgactcactatagggagaaggctTAAACTCCAATATACAATCATCCAC |
| CTCF_77 | CTCF_77-421-10F | aggaagagagGGTTTTTTAGTATTTGGTATGGAGGA |
|  | CTCF_77-746-T7R | cagtaatacgactcactatagggagaaggctAAAACACTACCATCTACAAAACCCA |
| CTCF_79 | CTCF_79-C-563-10F | aggaagagagGTTAGGTGGAGTTGTTTTTGGTG |
|  | CTCF_79-C-754-T7R | cagtaatacgactcactatagggagaaggctTCACTTCCAAACTCTTATCCTTATC |
| CTCF_84 | CTCF_84-C-479-10F | aggaagagagGTCGGAGAAGGAtGGAGTtGtT |
|  | CTCF_84-C-611-T7R | cagtaatacgactcactatagggagaaggctAACaAACGaAAaATTTTACaaaATACC |
| CTCF_85 | CTCF_85-C-440-10F | aggaagagagGGTTTGGTTTTTAGGAGAGGATTG |
|  | CTCF_85-C-755-T7R | cagtaatacgactcactatagggagaaggctAAACaATCaAAaaTCTAaATCaAAA |
| CTCF_94 | CTCF_94-C-514-10F | aggaagagagAATTGATAAAGGATGGGAGAATGTT |
|  | CTCF_94-C-740-T7R | cagtaatacgactcactatagggagaaggctACACCTACTACCTACCCTCCAAAAC |
| CTCF_101 | CTCF_101-C-507-10F | aggaagagagGGTTTTGAGGGAGGTGTTTATTTAG |
|  | CTCF_101-C-826-T7R | cagtaatacgactcactatagggagaaggctAAAAAACCCTATCCATATTCCTCAC |
| CTCF_103 | CTCF_103-C-813-10F | aggaagagagTAGTTAGAGTGTGGGTTAGGGTTTT |
|  | CTCF_103-C-537-T7R | cagtaatacgactcactatagggagaaggctACAACAACAACAAATCCAACAAC |
| CTCF_109 | CTCF_109-470-10F | aggaagagagGGTTAGTGTGGGTTGAATTATTTTG |
|  | CTCF_109-684-T7R | cagtaatacgactcactatagggagaaggctAAAACCAAACCTCATCTACCCTTTA |
| CTCF_113 | CTCF_113-552-10F | aggaagagagGTTTAGAGGAGAAAGGAATTTTTG |
|  | CTCF_113-847-T7R | cagtaatacgactcactatagggagaaggctCTAAAAACAACCCTCCTTTTACCC |
| BMP3 | BMP3-10F | aggaagagagTTTTAGTTGGTTTGGAGTTTAATTTT |
|  | BMP3-T7R | cagtaatacgactcactatagggagaaggctCAACCACAAAAAAAACAACCTACTC |
| NDRG4 | NDRG4-10F | aggaagagagAtGGGGAtGtTtTtGTAGGtT |
|  | NDRG4-T7R | cagtaatacgactcactatagggagaaggctATACCAAACCTAAAACTaATCC |

The reverse primer was tagged by a 31bp T7 promoter sequence for *in vitro* transcription and the forward primer was tagged by a 10bp tag-sequence to balance the PCR primer length.
